# Supplementary material for: Learning Transferable Push Manipulation Skills in Novel Contexts
Source: Front Neurorobot. 2021 Jun 7;15:671775. doi: 10.3389/fnbot.2021.671775 (PMC8215151; doi:10.3389/fnbot.2021.671775)
Supplement: Supplementary file 1 [file Data_Sheet_1.PDF]

Supplementary Material

1 SUPPLEMENTARY TABLES AND FIGURES

Table S1. Experimental parameter descriptions for prediction accuracy experiments.

Contact Model Generation		Condition Generation	
Distance	The cut-off distance δ_m for determining which contacts to keep when generating a contact model.	Number of Conditions to Generate	Number of push conditions to generate.
Lambda	The exponential drop-off rate λ_c used when calculating weights for contacts when generating a contact model.	Number of Environment Contacts	Number of environment contacts to place as part of the process of generating each push condition.
		Number of Samples When Generating Environment Contacts	Number of samples to take from the environment contact model when generating each environment contact.
**Motion Model Training**		**Ground Truth Generation**	
Number of Actions	Number of actions that the motion model will be trained for.	Number of Actions	Number of actions that will be simulated for each push condition.
Angle Range	Defines the range of angular velocities from which each action will be derived.	Angle Range	Defines the range of angular velocities from which each action will be derived.
Action Duration	Duration of push operation.	Action Duration	Duration of push operation.
Action Speed	Target speed of robot during push operation.	Action Speed	Target speed of robot during push operation.
Samples Per Action	Number of sample push simulations to carry out and record for each action.	Samples Per Action	Number of sample push simulations to carry out and record for each combination of action and push condition.
Object Mass	Object mass value or distribution from which the object mass will be sampled.	Object Mass	Object mass value or distribution from which the object mass will be sampled.
Object Coefficient of Friction	Object coefficient of friction value or distribution from which the object coefficient of friction will be sampled. Only present in initial experiments, coefficient of friction parametrisation was moved to ground plane to better represent real world conditions for later experiments.	Object Coefficient of Friction	Object coefficient of friction value or distribution from which the object coefficient of friction will be sampled. Only present in initial experiments, coefficient of friction parametrisation was moved to ground plane to better represent real world conditions for later experiments.
Ground Plane Coefficient of Friction	Ground plane coefficient of friction value or distribution from which the ground plane coefficient of friction will be sampled. Only present in later experiments as discussed above.	Ground Plane Coefficient of Friction	Ground plane coefficient of friction value or distribution from which the ground plane coefficient of friction will be sampled. Only present in later experiments as discussed above.
Number of Environment Contacts	Number of environment contacts to be recorded in conjunction with each push simulation.		
**Prediction Generation**			
Number of Environment Contacts	Number of environment contacts to use for each push condition when predicting the final object transform. Environment contacts are stored as part of each push condition following their placement in push condition generation.		
Environment Contact Kernels	Number of environment contact kernels to use for each push condition when predicting the final object transform. Kernels come from the PDRs that comprise the motion model in use.		
Manipulator Contact Kernels	Number of manipulator contact kernels to use for each push condition when predicting the final object transform. Kernels come from the PDRs that comprise the motion model in use.		

Table S2. Experimental parameters for evaluating the selection of contact and motion models.

Contact Model Generation		Condition Generation	
Distance	0.01	Number of Conditions to Generate	50
Lambda	100	Number of Environment Contacts	10
		Number of Samples When Generating Environment Contacts	10
**Motion Model Training**		**Ground Truth Generation**	
Number of Actions	3	Number of Actions	3
Angle Range	$[-10, 10]$	Angle Range	$[-10, 10]$
Action Duration (s)	4	Action Duration (s)	4
Action Speed (ms^{-1})	0.1	Action Speed (ms^{-1})	0.1
Samples Per Action	500	Samples Per Action	4
Object Mass (kg)	0.5	Object Mass (kg)	0.5
Object Coefficient of Friction	$U(0.15, 0.35)$	Object Coefficient of Friction	$U(0.15, 0.35)$
Number of Environment Contacts	10		
**Prediction Generation**			
Number of Environment Contacts	5		
Environment Contact Kernels	5000		
Manipulator Contact Kernels	500		

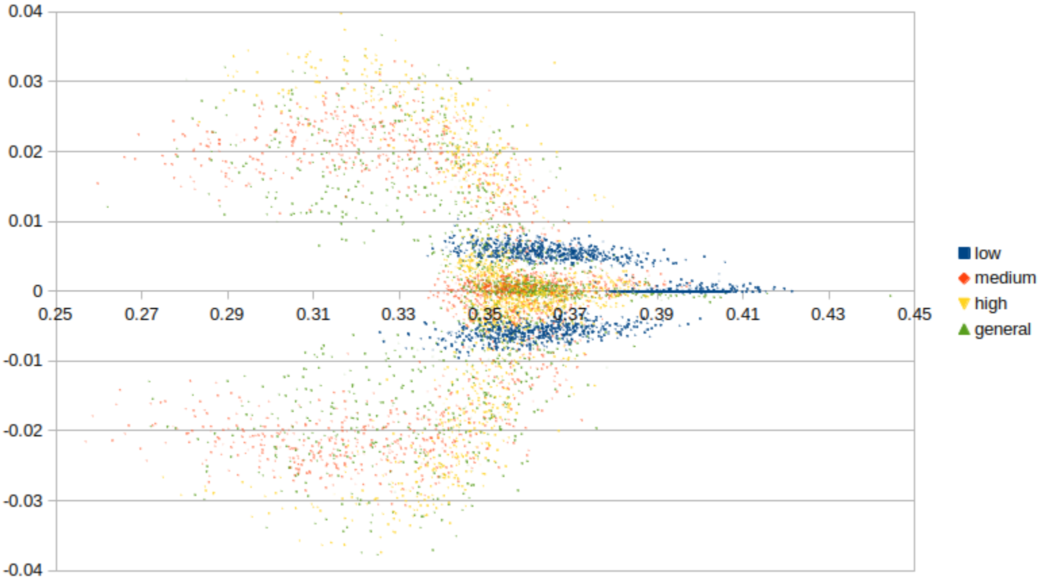

Figure S1. 2D plot of the learned motion models for a cube in environments with different friction conditions. The x and y axes in the plot are measured in metres. The $(0, 0)$ pose represents the initial pose of the object to be pushed and each dot represents the pose of the object after a push in a given condition. The different distributions are best demarked by their colour coding.

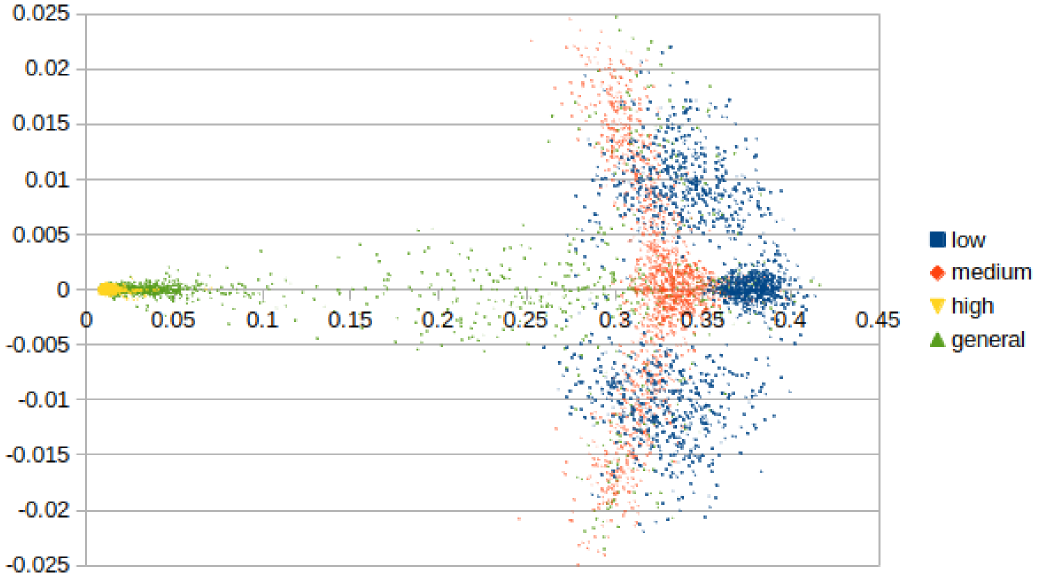

Figure S2. 2D plot of the learned motion models for a cube in environments with different mass conditions. The x and y axes in the plot are measured in metres. The $(0, 0)$ pose represents the initial pose of the object to be pushed and each dot represents the pose of the object after a push in a given condition. The different distributions are best demarked by their colour coding.

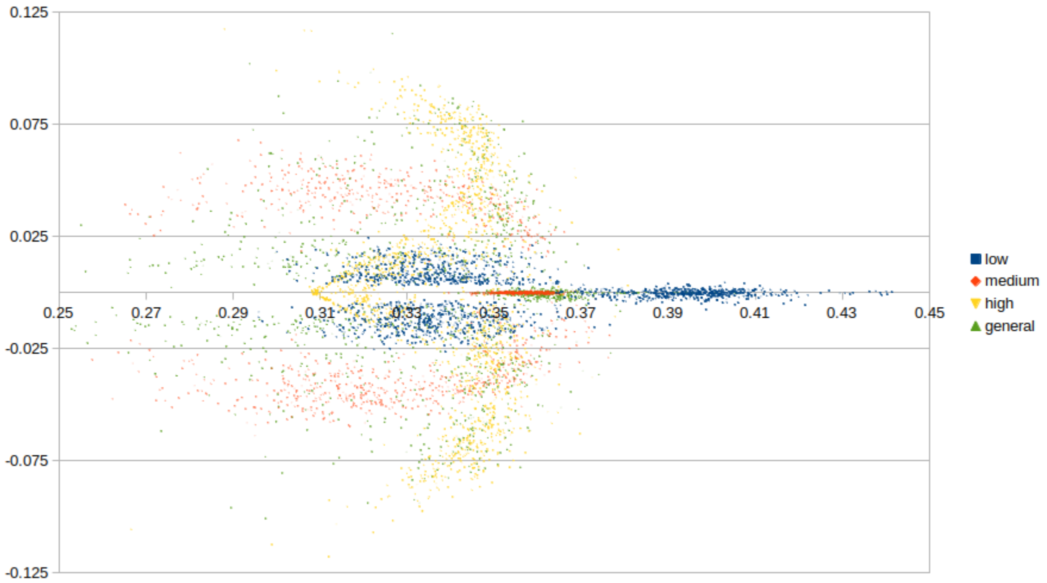

Figure S3. 2D plot of the learned motion models for a cylinder in environments with different friction conditions. The x and y axes in the plot are measured in metres. The $(0, 0)$ pose represents the initial pose of the object to be pushed and each dot represents the pose of the object after a push in a given condition. The different distributions are best demarked by their colour coding.

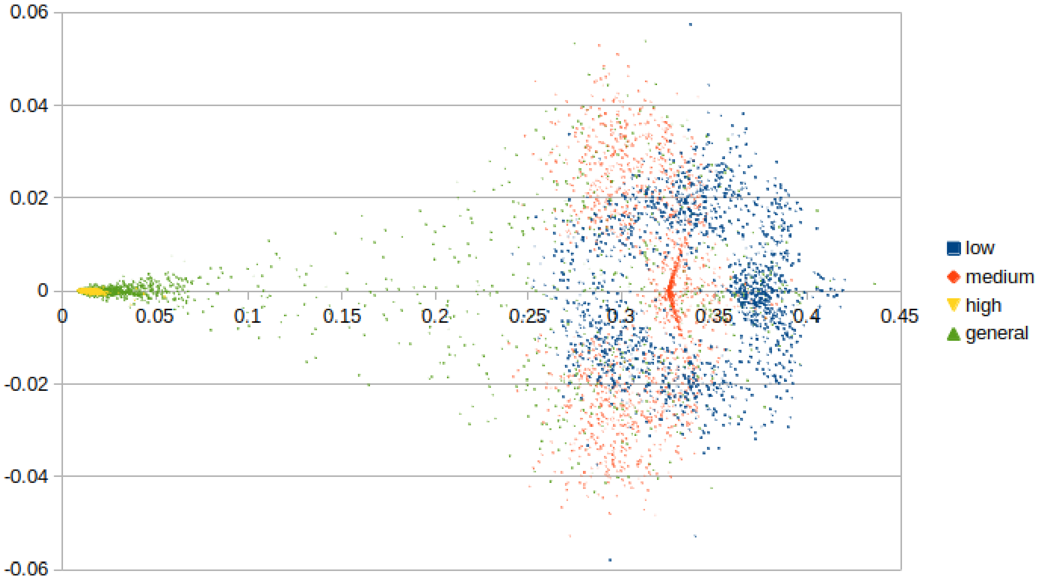

Figure S4. 2D plot of the learned motion models for a cylinder in environments with different mass conditions. The x and y axes in the plot are measured in metres. The $(0, 0)$ pose represents the initial pose of the object to be pushed and each dot represents the pose of the object after a push in a given condition. The different distributions are best demarked by their colour coding.
